# Association of natural teeth, dietary diversity, and nutritional status in elderly nursing home residents

**DOI:** 10.3389/fnut.2026.1770416

**Published:** 2026-05-04

**Authors:** Ke-hong Fang, Ye Lv, Xu-hui Zhang, Hui Liu, Bing-zhong Zhai, Yuan Yao, Li-ming Huang, Hong Xu

**Affiliations:** Department of Health Hazards Surveillance, Hangzhou Center for Disease Control and Prevention (Hangzhou Health Supervision Institution), Hangzhou, China

**Keywords:** dietary diversity, number of natural teeth, nursing home, nutritional status, older adults

## Abstract

**Objective:**

Tooth loss in older adults was associated with an elevated risk of malnutrition, which may be compounded by limited dietary diversity. This study aimed to assess the associations between the number of natural teeth, dietary diversity (DD), and nutritional status among elderly nursing home residents.

**Materials and methods:**

This cross-sectional study involved 1,710 nursing home residents aged ≥60 years from 26 facilities. Number of natural teeth, dietary diversity score (DDS), and nutritional status were assessed. Logistic regression and mediation analysis were used to examine the associations between the number of natural teeth, DD, and nutritional status, and the mediating role of DD.

**Results:**

Participants had a mean age of 83.28 ± 8.66 years. After adjusting for confounding variables, compared with participants with 0 natural teeth, those with ≥20 natural teeth showed a positive correlation with normal nutritional status (Odds Ratio [OR] = 4.29, 95% Confidence Interval [CI]:2.26–6.15). Additionally, compared with those with a DDS of ≤5, a DDS of 8 was associated with better nutritional status (OR = 2.11, 95%CI: 1.17–3.82). DD mediated the relationship between the number of natural teeth and nutritional status (*β* = 0.0075, 95%CI: 0.0041–0.0114).

**Conclusion:**

Present study indicated that older adults in nursing homes with a greater number of natural teeth tended to have better nutritional status. DD played a mediating role in this association, indirectly influencing the nutritional status of the elderly. These findings highlight the critical significance of the number of natural teeth in maintaining favorable nutritional status among the elderly. Meanwhile, they suggest that DD should be incorporated into the scope of attention when focusing on the nutritional status of the elderly.

## 1 Introduction

The global population is undergoing a rapid transition toward aging (1). Concomitant with this demographic shift, nutritional health among older adults has emerged as an increasingly prominent public health concern. Current estimates indicate that approximately one-quarter of adults aged 65 years and older are malnourished or at risk of malnutrition (2, 3). Specifically, the prevalence of malnutrition is 0.8–24.6% among community-dwelling older adults (4), and is even higher (5–50%) among those residing in nursing homes (5). In Chinese nursing homes, the incidence of malnutrition or the risk of malnutrition among older adults ranges from 8.2 to 30% (6).

Malnutrition not only accelerates the progression of chronic diseases but also significantly impairs the quality of life of older adults (6–8). Therefore, the timely and accurate identification of risk factors associated with malnutrition is a crucial step in preventing its occurrence and interrupting its progression.

Although numerous studies have explored the relationship between the number of natural teeth and nutritional status, empirical findings remain inconsistent. Some studies have suggested that a lower number of natural teeth is associated with a higher risk of malnutrition (9), whereas others have failed to establish a significant correlation (10). Furthermore, a previous meta-analysis was unable to confirm a definitive relationship between tooth loss and nutritional outcomes (11). These contradictory results suggest that the association between dental status and nutrition may not be a simple, direct link, but rather one governed by complex intermediary mechanisms that have yet to be fully elucidated.

Most existing studies have focused on bivariate associations and largely overlooked the integrated oral-dietary-nutritional pathway. Mechanistically, poor oral health (e.g., tooth loss) impairs masticatory function, which in turn restricts food choices. This often leads older adults to reduce their intake of hard-textured but nutrient-dense foods, such as vegetables, fruits, and meats. Such dietary shifts compromise DD, thereby increasing the risk of inadequate nutrient intake and ultimately culminating in malnutrition (12). This biologically plausible pathway provides a strong theoretical basis for considering DD as a potential mediator between the number of natural teeth and nutritional status. However, empirical evidence formally testing this mediating role remains scarce.

Furthermore, most existing research has focused on community-dwelling populations, whereas nursing home residents often exhibit poorer oral health and lower levels of dietary diversity (13–15). Within this vulnerable cohort, the relationships between natural teeth, DD, and nutritional status have not been sufficiently explored.

In view of the ongoing controversy regarding the association between natural tooth count and nutritional status, the limited evidence supporting the mediating effect of dietary diversity, and the relative lack of research focusing on older adults in nursing homes, this study aimed to examine the association between natural tooth count and nutritional status, as well as the mediating role of dietary diversity, among nursing home residents. The findings will provide a scientific basis for optimizing precision nutrition and health management strategies for older adults in nursing homes.

## 2 Materials and methods

### 2.1 Study design and study population

This study adopted a cross-sectional design based on the baseline data derived from the Nutrition Monitoring Program for Elderly Care Institutions in Hangzhou, an ongoing prospective cohort study conducted citywide from 2024 to 2026. While the overarching cohort study is designed for the longitudinal monitoring of health trajectories, the present analysis utilizes baseline data to examine the preliminary associations between oral health, dietary patterns, and nutritional status. Establishing these cross-sectional links is a fundamental prerequisite for elucidating potential mediation mechanisms before verifying causal relationships in future longitudinal assessments.

**Figure 1:**
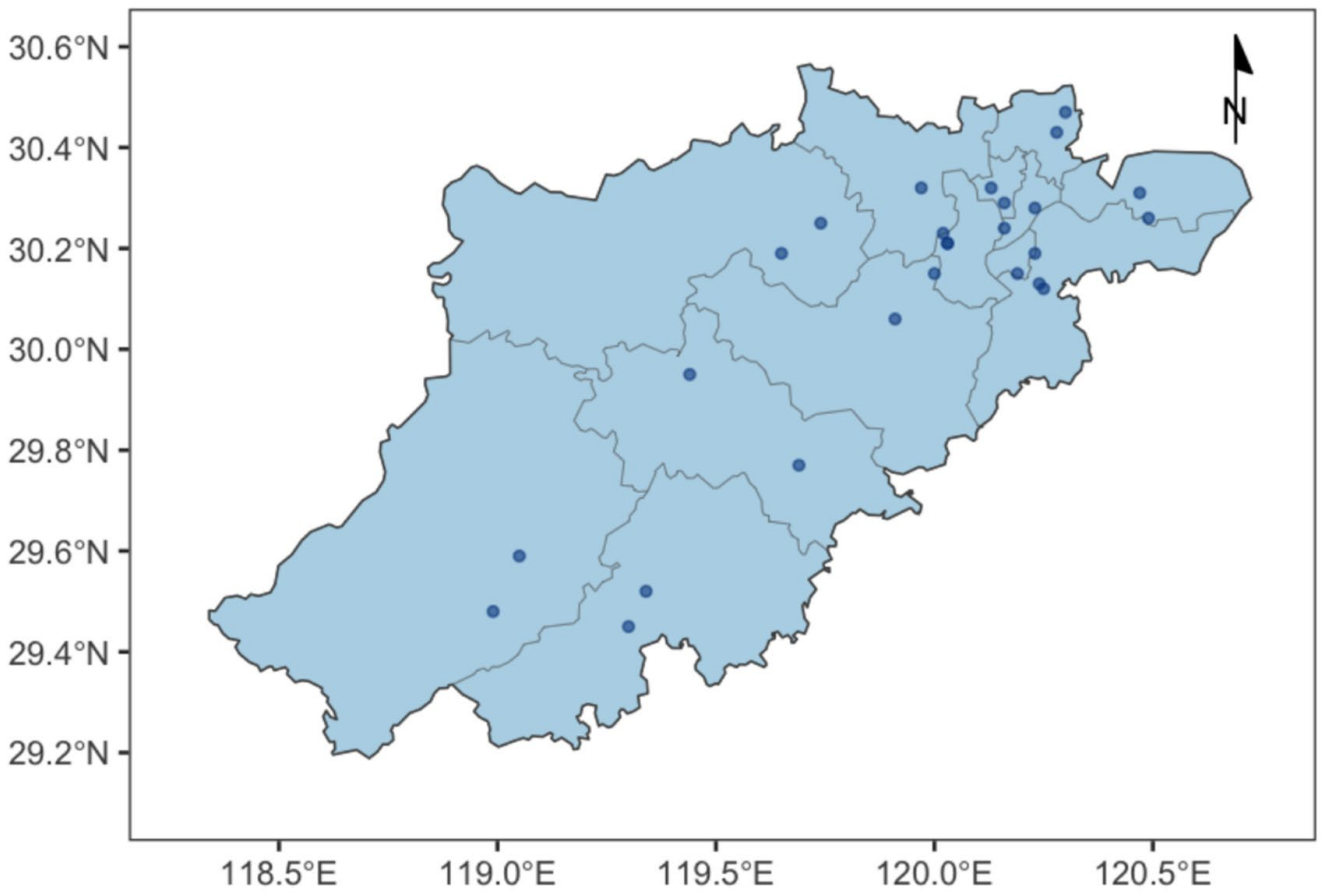
Spatial distribution of nursing homes in Hangzhou. The administrative division map data used in this study were obtained from Tianditu (National Platform for Common Geospatial Information Services) with the official approval number GS(2024)0650 (Available at: https://cloudcenter.tianditu.gov.cn/administrativeDivision).

**Figure 2:**
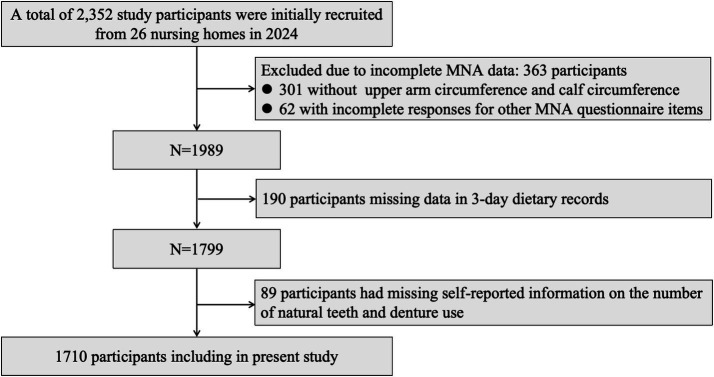
Participant inclusion diagram.

A multi-stage cluster sampling method was employed to recruit participants across all 13 districts and counties in Hangzhou, China. In the first stage, two nursing homes were randomly selected from each district, resulting in a total of 26 institutions. The geographical distribution of these participating nursing homes is illustrated in Figure 1. In the second stage, approximately 90 residents were recruited from each institution through coordination with nursing home healthcare staff. The inclusion criteria were: (1) aged 60 years or older; (2) resided in the institution for more than 6 months; and (3) capable of basic self-care and clear communication. The exclusion criteria were: (1) severe cognitive impairment or terminal illness that prevented cooperation; (2) missing Mini-Nutritional Assessment (MNA) data; (3) incomplete 3-day dietary records; or (4) missing self-reported information on natural tooth count or denture use. As detailed in the participant inclusion flowchart (Figure 2), 2,352 residents were initially recruited. After systematic screening based on the exclusion criteria, a final sample of 1,710 elderly individuals was included in the analysis. The study protocol was approved by the Ethics Committee of the Hangzhou Center for Disease Control and Prevention (Hangzhou Health Inspection Institute; No. 2024–41). Written informed consent was obtained from all participants prior to the survey.

### 2.2 Dietary survey and DDS

Individual dietary intake was assessed by trained staff using a consecutive three-day 24-h dietary recall combined with the food weighing method (including two weekdays and one weekend day). The dietary survey comprised two components: a canteen-based assessment and an individual-level consumption record.

In the canteen-based survey, investigators quantified all ingredients and condiments used for collective meal preparation. Given that breakfast was typically prepared the previous evening, researchers accessed the kitchens both on the night before and the morning of the survey to weigh all raw materials designated for that day’s breakfast service. An identical weighing protocol was applied to lunch and dinner preparations.

For individual consumption data, investigators recorded the portion sizes of various foods consumed by each participant during canteen meals, with reference to the standard menus provided by the canteens (including staple foods). For any externally provided foods consumed by the subjects, investigators interviewed them to document the food names, ingredient compositions, estimated weights, and consumption times.

The DDS was developed according to the Dietary Guidelines for Chinese Residents (2022) (15), which provide recommendations for the consumption of 10 food groups, including staple foods (cereals, tubers, and beans), vegetables, fruits, eggs, aquatic products, meat and poultry, soybeans and nuts, milk and dairy products, salt, and oil. As salt and oil are essential parts of the Chinese diet, they were excluded when assessing DDS (16). A score of 1 point was awarded to each food group if at least one food item from that category was consumed during the dietary survey period; however, consumption of multiple different food items within the same category did not yield additional points. For mixed dishes (e.g., pork and cabbage dumplings), investigators decomposed it into specific ingredients (e.g., pork, cabbage, and flour) based on standardized recipes provided by the canteens. Each ingredient was then assigned to its corresponding food group (e.g., pork to meat and poultry, cabbage to vegetables, and flour to staple foods) to ensure the accuracy of the DDS calculation. No minimum intake threshold was implemented during the scoring process, resulting in a cumulative DDS ranging from 0 to 8 points. For analytical purposes, the DDS was further stratified into four categories (≤5, 6, 7, and 8) based on the quantile distribution within the study population.

### 2.3 Self-reported dentition status

Dentition status was assessed through standardized interviews rather than clinical examination by a dentist, where participants were asked, “How many natural teeth do you have (including third molars)?” and “Do you wear dentures?”. Dentition status was divided into 4 groups based on the number of natural teeth: 0, 1–9, 10–19 and ≥20 (9).

### 2.4 Nutritional status assessment

The MNA is a screening instrument designed to identify the risk of malnutrition in older adults. It comprises 18 items, covering general health status, weight loss, dietary intake, subjective health and nutrition evaluations. According to the MNA total scores, nutritional status was categorized as follows: ≥ 24 points indicated normal nutritional status, 17–23.5 points indicated a risk of malnutrition, and <17 points indicated malnutrition (17, 18).

### 2.5 Covariates definition

All covariates were collected by face-to-face interview. Sociodemographic characteristics included age (as a continuous variable), gender (male or female), level of education (primary school or less, middle school, college and above), type of residence (rural or urban), married status (unmarried or married), monthly income (<3,000 yuan, 3,000–5,000 yuan and >5,000 yuan), smoking status (no or yes), drinking status (no or yes), regular exercise (no or yes).

### 2.6 Statistics

The characteristics of the study participants were described according to their nutritional status. Differences among the two groups of nutritional status were tested by χ^2^ test for categorical variables and t test for continuous variables. Potential confounders were selected based on previously published literature and their biological plausibility. Logistic regression was used to analyze the correlation between the DD, dentition status and nutritional status, adjusting for sociodemographic characteristics (age, gender, residence, education, marital status, and monthly income) and lifestyle factors (smoking, drinking, and regular exercise) in a stepwise manner. Four models were constructed: Model 1 was unadjusted; Model 2 adjusted for sociodemographic factors; Model 3 additionally adjusted for lifestyle factors; and Model 4 further adjusted for denture use. The mediation analysis was performed to examine whether DDS mediated the effect of natural teeth on nutritional status using the PROCESS macro (Model 4) in SPSS. To test the significance of the indirect (mediating) effect, we employed bias-corrected percentile bootstrapping (5,000 samples). All statistical analyses were conducted using IBM SPSS Statistics version 25.0 (SPSS Inc., Chicago, IL, United States), and visualization was performed with GraphPad Prism version 9 and R version 4.4.1. The level of statistical significance was set at *p* < 0.05.

## 3 Results

### 3.1 Characteristics of the participants

The demographic characteristics of the participants, stratified by nutritional status, are presented in Table 1. A total of 1,710 older adults residing in nursing homes were included in the present study, with a mean age of 83.28 years (SD = 8.66), among whom 647 (37.84%) were males. The MNA score of the study participants was 21.33 ± 3.74. The proportions of participants with malnutrition, risk of malnutrition and normal nutritional status were 13.39, 57.02 and 29.59%, respectively. The DDS of the malnutrition group, risk of malnutrition group and normal nutritional status group were 6.20 ± 1.11, 6.57 ± 1.01 and 6.80 ± 0.97, respectively, while the number of natural teeth were 14.05 ± 9.27, 15.54 ± 9.24 and 17.78 ± 9.46, respectively. There were statistically significant differences in the distribution of the number of natural teeth and DD among participants with different nutritional statuses (*p* < 0.01).

**Table 1:** Characteristics of participants with nutritional status.

Variable	Total	Malnutrition	Risk of malnutrition	Normal nutritional	*p*
No. of participants (%)	1710	229(13.39)	975 (57.02)	506(29.59)	
Age, years (mean [SD])	83.28 ± 8.66	83.44 ± 9.19	83.25 ± 8.55	83.26 ± 8.63	0.9523
Gender (%)					0.5034
Male	647(37.84)	79(34.50)	377(38.67)	191(37.75)	
Female	1,063(62.16)	150(65.50)	598(61.33)	315(62.25)	
Education (%)					<0.0001
Primary school or less	800(46.78)	158(69)	475(48.72)	167(33.00)	
Middle school	627(36.67)	56(24.45)	350(35.90)	221(43.68)	
College and above	283(16.55)	15(6.55)	150(15.38)	118(23.32)	
Type of residence (%)					<0.0001
Urban	901(52.69)	80(34.93)	494(50.67)	327(64.62)	
Rural	809(47.31)	149(65.07)	481(49.33)	179(35.38)	
Marital status (%)					0.0044
Unmarried	760(44.44)	110(48.03)	400(41.03)	250(49.41)	
Married	950(55.56)	119(51.97)	575(58.97)	256(50.59)	
Monthly income, yuan (%)					<0.0001
<3,000	637(37.25)	136(59.39)	383(39.28)	118(23.32)	
3,000–5,000	425(24.85)	52(22.71)	248(25.44)	125(24.70)	
>5,000	648(37.89)	41(17.90)	344(35.28)	263(51.98)	
Smoking (%)					0.3187
No	1,610(94.15)	220(96.07)	912(93.54)	478(94.47)	
Yes	100(5.85)	9(3.93)	63(6.46)	28(5.53)	
Drinking (%)					<0.0001
No	1,627(95.15)	228(99.56)	932(95.59)	467(92.29)	
Yes	83(4.85)	1(0.44)	43(4.41)	39(7.71)	
Regular exercise (%)					<0.0001
No	820(47.95)	171(74.67)	475(48.72)	174(34.39)	
Yes	890(52.05)	58(25.33)	500(51.28)	332(65.61)	
Denture (%)					<0.0001
No	1,085(63.45)	172(75.11)	635(65.13)	278(54.94)	
Yes	625(36.55)	57(24.89)	340(34.87)	228(45.06)	
DDS (mean [SD])	6.58 ± 1.03	6.20 ± 1.11	6.57 ± 1.01	6.80 ± 0.97	<0.0001
DD (%)					<0.0001
≤5	253(14.80)	69(30.13)	132(13.54)	52(10.28)	
6	597(34.91)	68(29.69)	394(40.41)	135(26.68)	
7	446(26.08)	60(26.20)	207(21.23)	179(35.38)	
8	414(24.21)	32(13.97)	242(24.82)	140(27.67)	
Number of natural teeth (mean [SD])	16.00 ± 9.39	14.05 ± 9.27	15.54 ± 9.24	17.78 ± 9.46	<0.0001
Number of natural teeth (%)					<0.0001
0	181(10.58)	31(13.54)	105(10.77)	45(8.89)	
1–9	276(16.14)	54(23.58)	164(16.82)	58(11.46)	
10–19	502(29.36)	65(28.38)	290(29.74)	147(29.05)	
≥20	751(43.92)	79(34.50)	416(42.67)	256(50.59)	
MNA score	21.33 ± 3.74	14.24 ± 2.10	21.01 ± 1.85	25.15 ± 1.09	<0.0001

### 3.2 Proportion of various food intake among the participants

**Figure 3:**
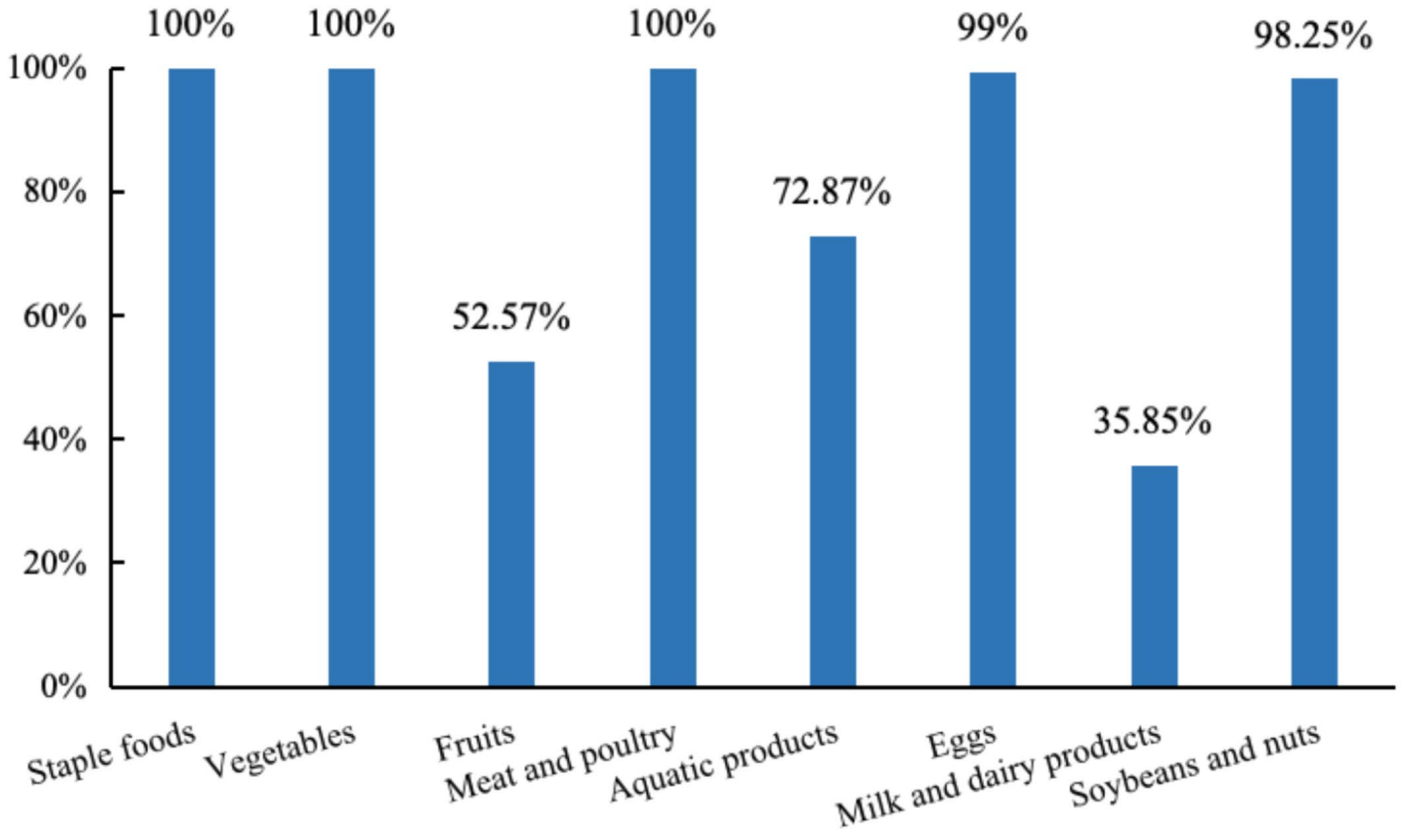
Proportion of various food intake among the participants.

As shown in Figure 3, all participants consumed staples, vegetables, meat and poultry during the 3-day survey, with the intake proportions of fruits, aquatic products, eggs, milk and dairy products, soybeans and nuts being 52.57, 72.87, 99.30, 35.85, and 98.25%, respectively.

### 3.3 MNA scores by number of natural teeth and DD

**Figure 4:**
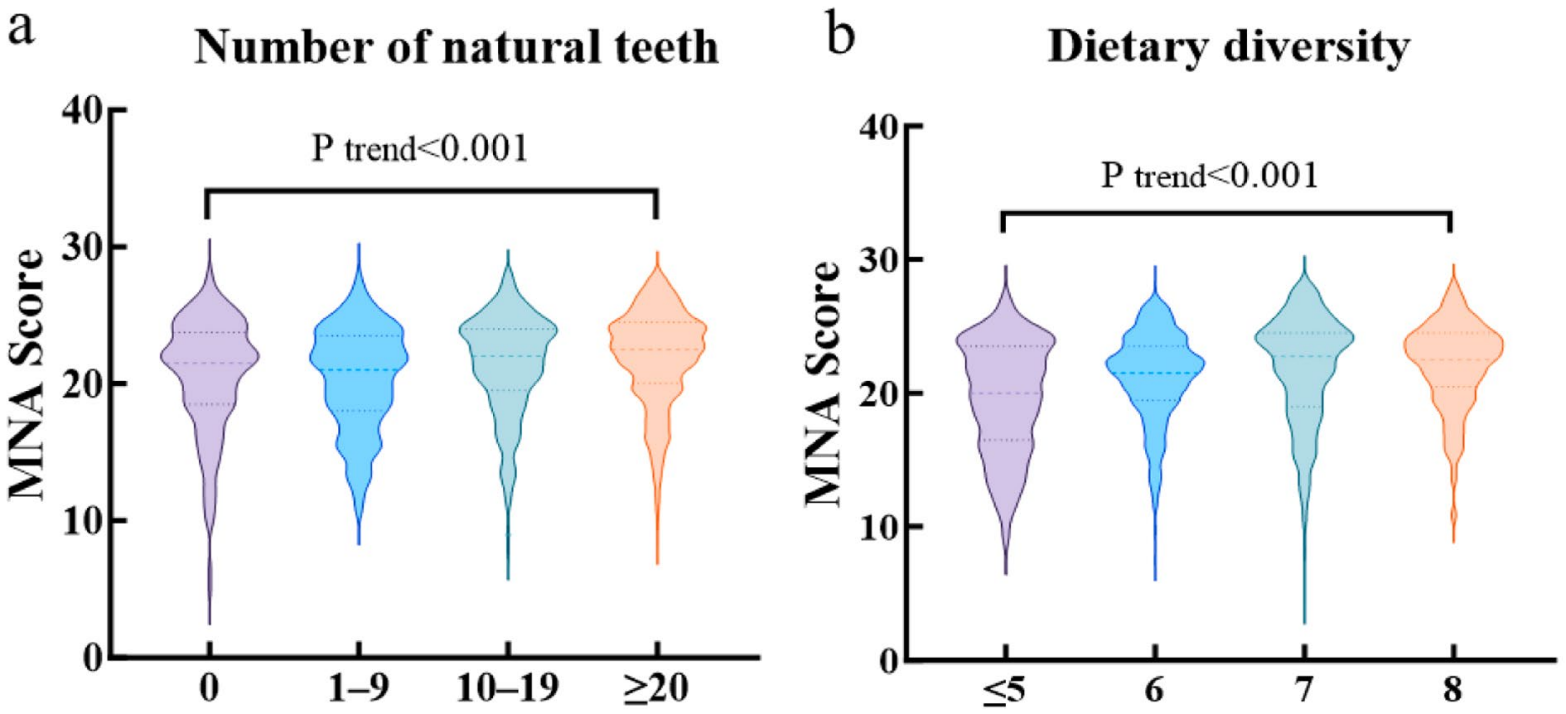
MNA score of participants by number of natural teeth and DD. **(a)** MNA score of participants with different number of natural teeth; **(b)** MNA score of participants with different DDS.

Figure 4 shows the MNA scores of elderly participants stratified by the number of natural teeth and DD. The MNA scores of elderly individuals with 0, 1–9, 10–19, and ≥20 natural teeth were 20.62 ± 4.17, 20.37 ± 3.74, 21.33 ± 3.75, and 21.85 ± 3.54, respectively. An upward trend in MNA scores was observed with the increase in the number of natural teeth, and the difference was statistically significant (p trend < 0.001). Similarly, MNA scores increased progressively with the elevation of DDS. The MNA scores of elderly participants with a DDS of ≤5, 6, 7, and 8 DD were 19.64 ± 4.18, 21.22 ± 3.35, 21.71 ± 4.13, and 22.10 ± 3.20, respectively.

### 3.4 The association between number of natural teeth and nutrition status

**Figure 5:**
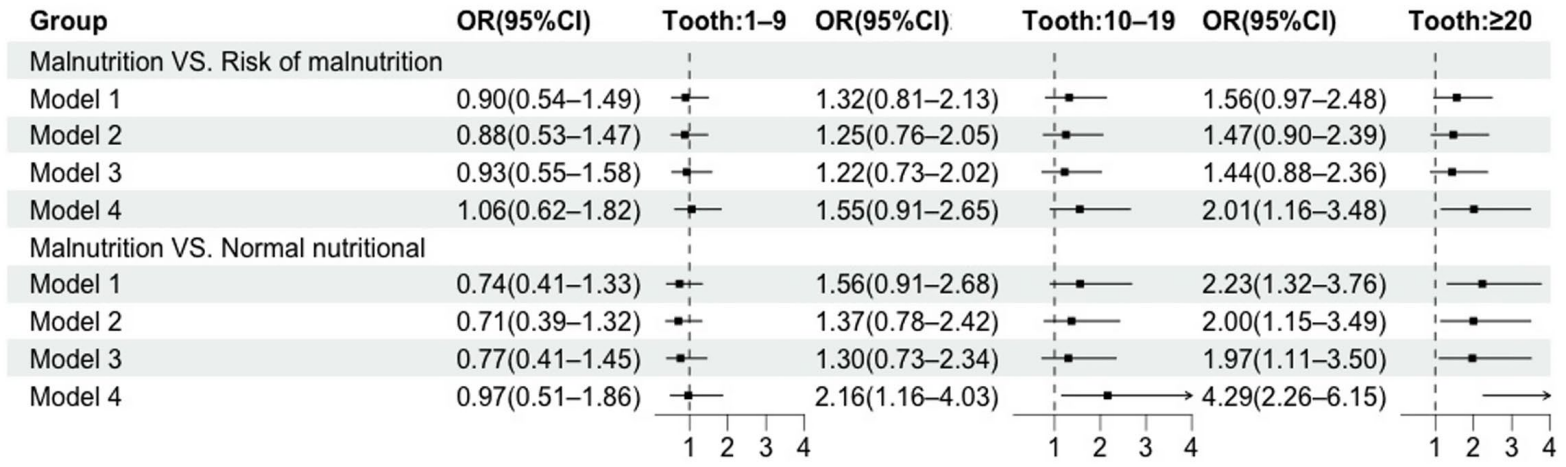
Forest plot depicting OR and 95% CI for association between the number of natural teeth and nutritional status.

Figure 5 presents the association between the number of natural teeth and nutritional status. In the crude model, only older adults with ≥20 natural teeth had a higher likelihood of normal nutritional status compared with those without natural teeth. After adjusting for covariates, model 4 showed that older adults with 10–19 natural teeth had an adjusted OR of 2.16 (95% CI: 1.16–4.03) for normal nutritional status, while those with ≥20 natural teeth exhibited a higher adjusted OR of 4.29 (95%CI: 2.26–6.15).

Model 1 was unadjusted. Model 2 was adjusted for age, gender, type of residence, education, household income, marital status, and monthly income. Model 3 was additionally adjusted for smoking, drinking and exercise. Model 4 was additionally adjusted for denture.

### 3.5 The association between DD and nutrition status

**Figure 6:**
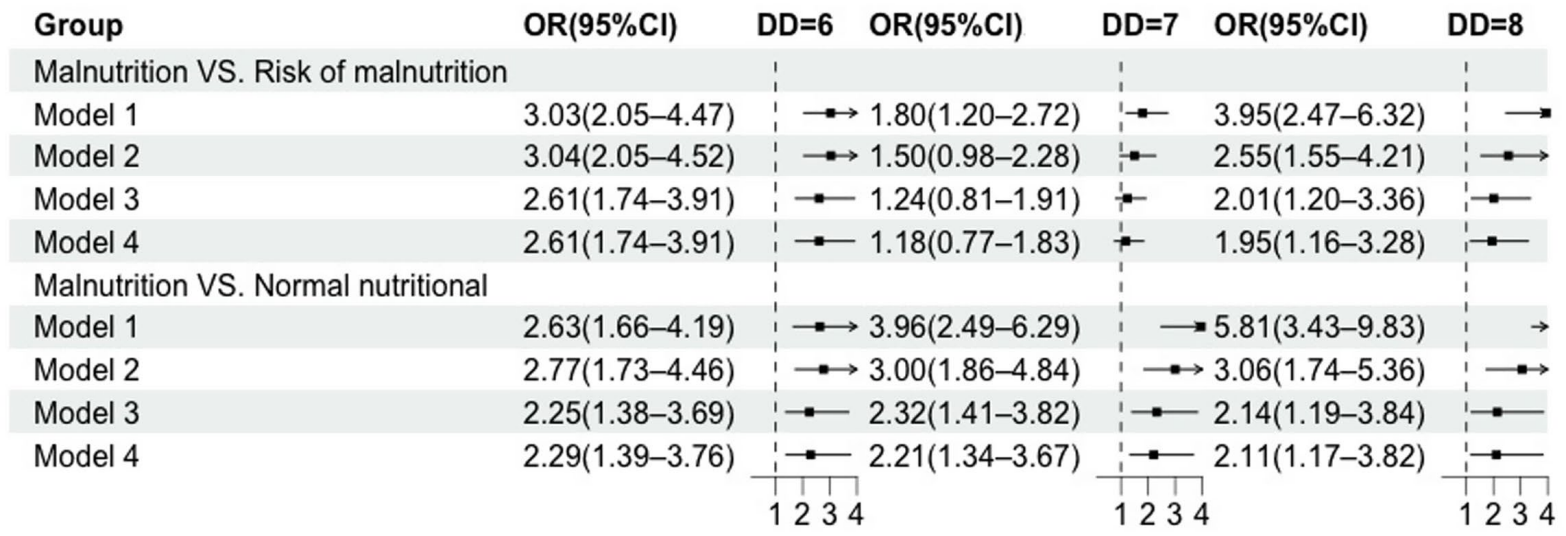
Forest plot depicting OR and 95% CI for association between the DD and nutritional status.

Figure 6 illustrates the association between DDS and nutritional status. Compared with older adults with a DDS of ≤5, those with scores of 6, 7, and 8 exhibited a positive correlation with normal nutritional status. The association between DDS and nutritional status remained significant in Model 4. Specifically, using DDS ≤ 5 as the reference, the adjusted ORs (95% CI) for achieving normal nutritional status were 2.29 (1.39–3.76) for DDS 6, 2.21 (1.34–3.67) for DDS 7, and 2.11 (1.17–3.82) for DDS 8.

Model 1 was unadjusted. Model 2 was adjusted for age, gender, type of residence, education, household income, marital status, and monthly income. Model 3 was additionally adjusted for smoking, drinking and exercise. Model 4 was additionally adjusted for denture.

### 3.6 The mediating role of the DDS between number of natural teeth and nutrition status

**Figure 7:**
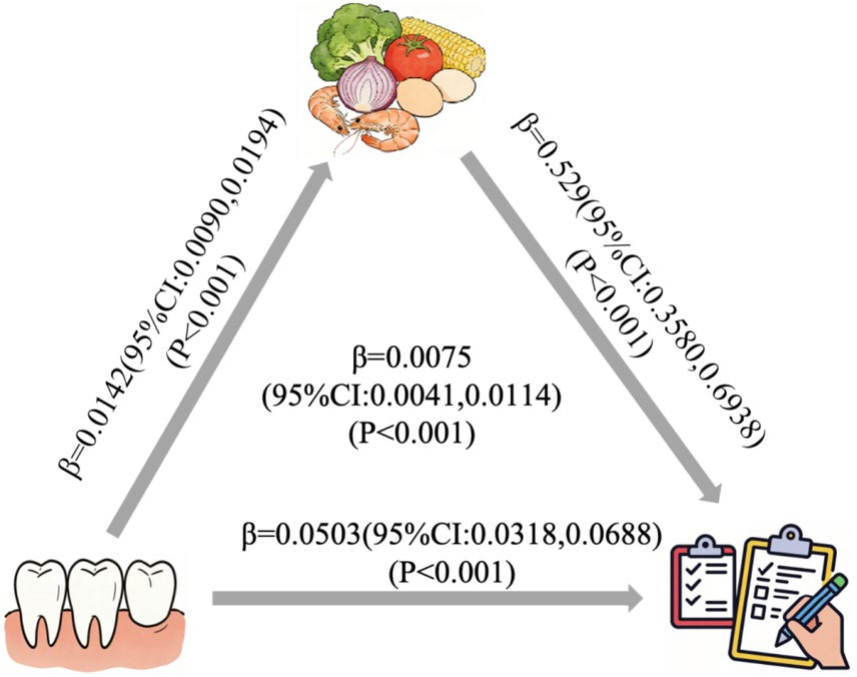
Path coefficient plots for the number of natural teeth, DDS, and nutrition status.

The study utilized mediation analysis to investigate the potential pathways of DD. Figure 7 illustrates the correlation between the number of natural teeth and nutritional status with DD as the mediator. Results of the mediation model analysis revealed that a greater number of natural teeth was were associated with higher DD (*β* = 0.0142, *p* < 0.001), and DD was positively correlated with nutritional status (*β* = 0.5259, *p* < 0.001). As shown in Table 2, the indirect association (*β* = 0.0075, 95%CI: 0.0041–0.0114), direct association (*β* = 0.0503, 95%CI: 0.0318–0.0688), and total association (*β* = 0.0557, 95%CI: 0.0392–0.0703) of the number of natural teeth on nutritional status were all statistically significant. The direct and indirect pathways accounted for 87.00 and 13.00% of the total association, respectively.

**Table 2:** Breakdown of total, direct, and indirect association.

Path	Efficiency value	SE	95%CI		Effect size
			LLCI	ULCI	
Total association	0.0577	0.0094	0.0392	0.0703	
Direct association	0.0503	0.0094	0.0318	0.0688	87.00%
Indirect association	0.0075	0.0018	0.0041	0.0114	13.00%

SE, Standard Error. LLCI, Lower Limit Confidence Interval. ULCI, Upper Limit Confidence Interval.

### 3.7 The association between the food intake, number of natural teeth and nutritional status

The MNA scores at levels of food intake are shown in Supplementary Figure S1. Participants who consumed fruits had a significantly higher MNA score compared with non-consumers (22.13 ± 3.28 vs. 20.44 ± 4.01, *p* < 0.05). Similarly, scores were higher among consumers of aquatic products (21.59 ± 3.63 vs. 20.62 ± 3.94), and milk and dairy products (21.61 ± 3.84 vs. 21.17 ± 3.68), with these differences also being statistically significant (*p* < 0.05).

The associations between the number of natural teeth and food intake are illustrated in Supplementary Figure S2. A greater number of natural teeth was associated with significantly higher odds of consuming fruits (OR = 1.45, 95% CI: 1.01–2.09), aquatic products (OR = 3.72, 95% CI: 2.49–5.57) and dairy products (OR = 1.85, 95% CI: 1.18–2.91). As illustrated in Supplementary Figure S3, in adjusted models, the intake of fruits (OR = 2.50, 95% CI: 1.38–3.06) and aquatic products (OR = 1.81, 95% CI: 1.26–2.60) was associated with normal nutritional status. In contrast, no significant association was found between the intake of milk and dairy products and nutritional status.

## 4 Discussion

This study focused on older adults in nursing homes, a group at high risk of malnutrition, to systematically explore the association between the number of natural teeth and nutritional status, while providing preliminary evidence on the potential mediating role of dietary diversity.

Our findings indicate that older adults retaining 20 or more natural teeth exhibit a significantly superior nutritional status. This conclusion aligns closely with international geriatric health initiatives, particularly Japan’s “8,020 Campaign,” which emphasizes the necessity of maintaining at least 20 functional teeth at age 80 to ensure adequate masticatory efficiency and quality of life (19). Furthermore, both the Otassha Study in Japan and research conducted within Western dietary contexts have demonstrated that tooth loss restricts the intake of high-fiber foods, high-quality proteins, and essential micronutrients (20, 21). The results of the present study are highly consistent with this global evidence and relevant systematic reviews (11, 22, 23), collectively highlighting a significant negative correlation between tooth count and the risk of malnutrition.

Several potential mechanisms may explain the association between dentition status and malnutrition. At the behavioral level, tooth loss impairs masticatory efficiency, triggering “food avoidance behaviors.” Older adults often reduce their consumption of chewing-intensive foods, such as fibrous vegetables, fruits, and high-quality proteins, in favor of soft, processed carbohydrates that are less nutrient-dense. This dietary imbalance leads to “hidden hunger,” a condition where total energy intake may appear stable while a severe deficit of essential micronutrients occurs (24). At the biological level, the lack of sufficient mechanical breakdown of food in the oral cavity causes larger particles to enter the gastrointestinal tract. This restricts the accessibility of digestive enzymes to nutrients, particularly proteins and complex carbohydrates, thereby reducing overall digestive and absorptive efficiency (25–27). Such an impact is especially critical for older adults, whose gastrointestinal functions are typically already in decline.

Mediation analysis further confirms that a decline in dietary diversity serves as one of the central pathways through which tooth loss translates into malnutrition. Consistent with existing literature (28–30), as well as the present study, older adults with missing teeth tend to forgo nutrient-dense foods such as nuts and meat; this shift in dietary patterns directly restricts the sources of micronutrient intake. Our findings support empirical evidence from the Chinese Longitudinal Healthy Longevity Study, the National Health and Nutrition Examination Survey (9, 29) and align with clinical observations from Iran and Ethiopia regarding DDS (31–34). A more profound clinical implication is that the deficiency of high-quality proteins and antioxidants, resulting from insufficient dietary diversity, impairs muscle protein synthesis, thereby elevating the risk of sarcopenia and frailty (35, 36). This progression from oral dysfunction to systemic physical decline underscores the fact that oral health is a fundamental cornerstone for maintaining functional independence and living ability in the elderly population.

The present study further refined the aforementioned associations at the food group level, revealing that individuals with a higher number of natural teeth exhibited a significant advantage in the intake of fruits and aquatic products. Antioxidants found in fruits contribute to enhancing immune function (37), while aquatic products provide high-bioavailability protein that is essential for the maintenance of muscle mass (38). Notably, although the intake of dairy products did not reach statistical significance in this study, this may be attributed to the generally low level of dairy consumption among the Chinese elderly population, with only 2% meeting the recommended intake standards (39). Given the specific demand for soft nutrient sources in later life, dairy products, as a vital source of high-quality protein, maintain substantial potential for nutritional interventions in populations with missing teeth (40). Future intervention strategies should prioritize oral preventive care along with the development of texture-modified functional foods to bridge the nutritional gaps resulting from an insufficiency of natural teeth.

The innovation of this study lies in its focus on nursing home residents, a high-risk population, clarifying the association between the number of natural teeth and nutritional status, and the mediating role of dietary diversity. This provides a scientific basis for nutritional interventions and oral health management in the elderly. The study results suggest that nursing homes should strengthen oral health screening for older adults, promptly provide restorative treatment (such as denture fitting) for those with tooth loss, and simultaneously emphasize enhancing dietary diversity by developing personalized dietary plans to improve the nutritional status of older adults. However, there are some limitations: First, this study adopted a cross-sectional design. Although it revealed the associations and mediating mechanisms between variables, it cannot establish a direct causal relationship among the number of natural teeth, dietary diversity, and nutritional status. The mediation analysis, in particular, should be interpreted with caution as it assumes a causal direction that cannot be fully verified without temporal sequencing. Therefore, these results should be viewed as preliminary evidence of a potential pathway, and future prospective cohort studies will be necessary to verify these temporal sequences and causal pathways. Second, this study was limited by the availability of existing data, and some important potential confounders, such as chronic diseases, actual chewing efficiency, and the functional status of the elderly, were not included in the multivariable models. These factors are known to be closely related to both oral health and nutritional status (41, 42). Although we adjusted for age, lifestyle factors, and the use of dentures as a partial proxy for chewing restoration, the absence of these variables may introduce residual confounding. Consequently, the associations reported in this study should be interpreted with caution, as they may be partially influenced by these unmeasured health-related factors. Third, the number of natural teeth was assessed by self-report rather than clinical examination by a dentist, which may have introduced reporting bias. Nevertheless, this method was chosen for its feasibility in a citywide investigation of 26 nursing homes. It is noteworthy that previous validation studies have demonstrated high agreement (correlation coefficient = 0.97) between self-reported and clinically determined tooth counts in older adults (43), supporting the validity of self-reported dentition status as a reliable proxy in large-scale epidemiological investigations (44). Finally, the sample for this study was drawn from nursing homes in a specific region of China, which might limit the generalizability of its results. Future research could further extend to broader geographical areas and different types of nursing homes to enhance the external validity of the study’s conclusions.

## 5 Conclusion

In summary, this study clarifies the significant positive correlation between the number of natural teeth and nutritional status among older adults in Chinese nursing homes, and confirms the critical mediating role of dietary diversity in this relationship.

These findings not only offer a new perspective for a deeper understanding of the complex mechanisms linking oral health and nutritional status but also provide important scientific evidence for nursing homes to formulate comprehensive and precise nutritional care strategies for the elderly. Such initiatives should be grounded in oral health management and centered on the enhancement of dietary diversity. By implementing integrated preventive measures that link oral health maintenance with precision dietary management, residential care facilities can more effectively prevent and alleviate malnutrition among the elderly population, thereby establishing a fundamental cornerstone for promoting healthy aging and maintaining functional independence.

## Data Availability

The raw data supporting the conclusions of this article will be made available by the authors, without undue reservation.
