# Supplementary material for: Association of natural teeth, dietary diversity, and nutritional status in elderly nursing home residents
Source: Front Nutr. 2026 May 4;13:1770416. doi: 10.3389/fnut.2026.1770416 (PMC13180571; doi:10.3389/fnut.2026.1770416)
Supplement: Supplementary file 1 [file Image_1.PDF]

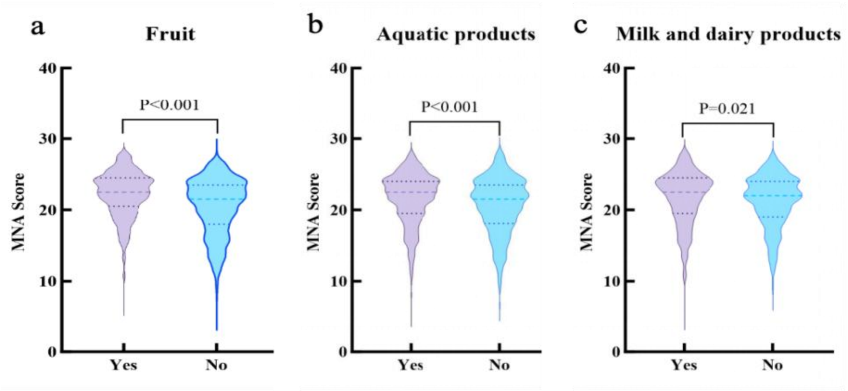

a. MNA Score of participants by intake levels of fruits; b. MNA Score of participants by intake levels of aquatic products; c. MNA Score of participants by intake levels of milk and dairy products

Supplementary Figure 1. MNA Score of participants by intake levels of various food

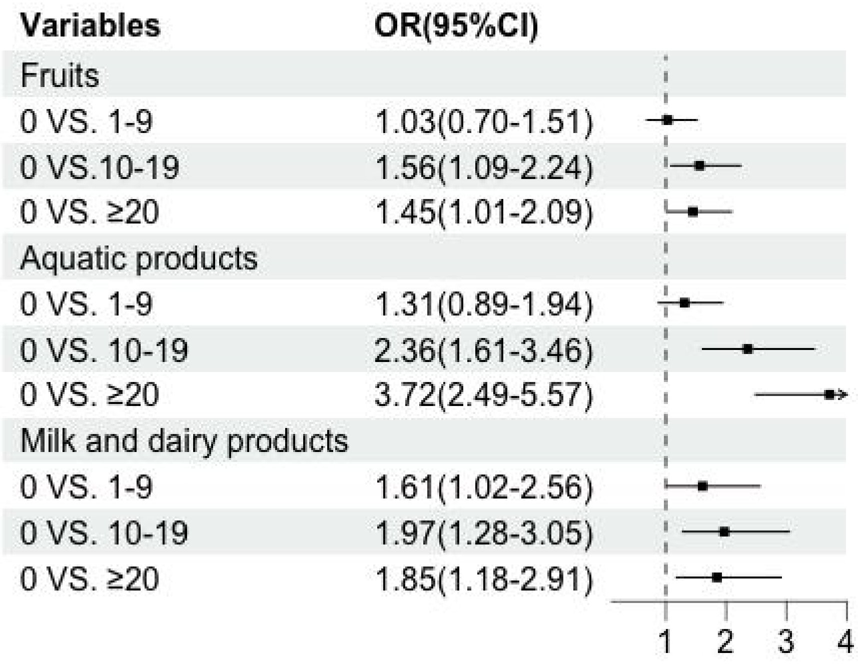

Supplementary Figure 2. The associations between the number of natural teeth and food intake

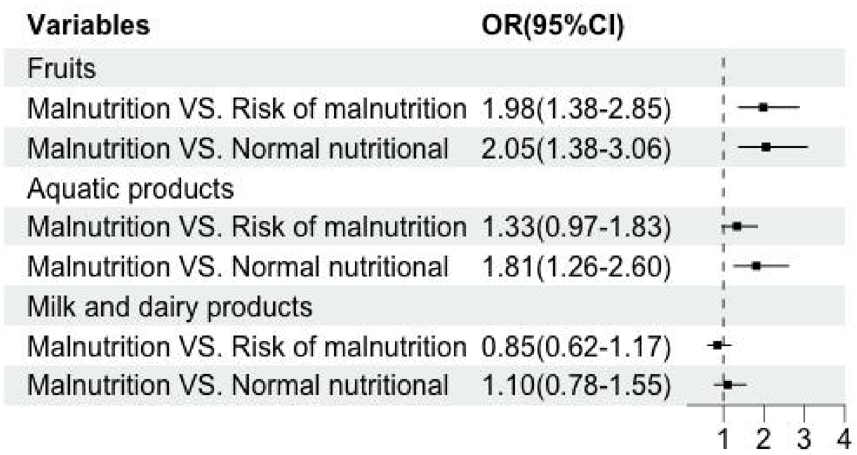

Supplementary Figure 3. The associations between the food intake and nutritional status
